# Lipid Histiocytosis of the Gallbladder Neck Lymph Node

**DOI:** 10.1155/2016/1061507

**Published:** 2016-10-26

**Authors:** Adriana Handra-Luca, Mohamed Habib Ben Romdhane, Beate Katharina Straub

**Affiliations:** ^1^APHP, Service d'Anatomie Pathologique, Bobigny, France; ^2^Université Paris Nord Sorbonne Cité, Bobigny, France; ^3^APHP, Service de Radiologie, Bobigny, France; ^4^Institute of Pathology, University Clinic Heidelberg, Heidelberg, Germany; ^5^Institute of Pathology, University Mainz, Mainz, Germany

## Abstract

Lipid histiocytosis of the gallbladder neck lymph node is rarely reported nowadays. Two obese patients presented with gallbladder lithiasis detected on CT scan. The treatment consisted in coelioscopic cholecystectomy. Microscopy revealed subacute/chronic lithiasic cholecystitis and foci of vacuolated cells in the gallbladder neck lymph node. These cells were positive for CD68, CD31, S100 protein, and adipophilin and negative for cytokeratin and Alcian blue. In conclusion, we report lymph node lipid histiocytosis diagnosed microscopically after cholecystectomy. While such lesions may remain unidentified on imaging procedures, the microscopic analysis may require special stains and immunohistochemistry for ruling out adenocarcinoma metastasis.

## 1. Introduction

In the 1960s, lipid deposits in the gallbladder neck lymph node (Mascagni-Lund), known also as lipophagic reactions/granuloma or lipid adenopathy, were reported in approximatively one-third of adults as an incidental finding at postmortem examination or on surgical resection specimen. The adjacent gallbladders were normal or showed cholesterolosis and/or calculous cholecystitis [1–3]. There are few recent data on the histogenesis of such lesions.

Here we report two cases of lymph node lipid histiocytosis diagnosed incidentally on cholecystectomy specimens resected for gallbladder lithiasis in obese patients.

## 2. Case Report

**Figure 1:**
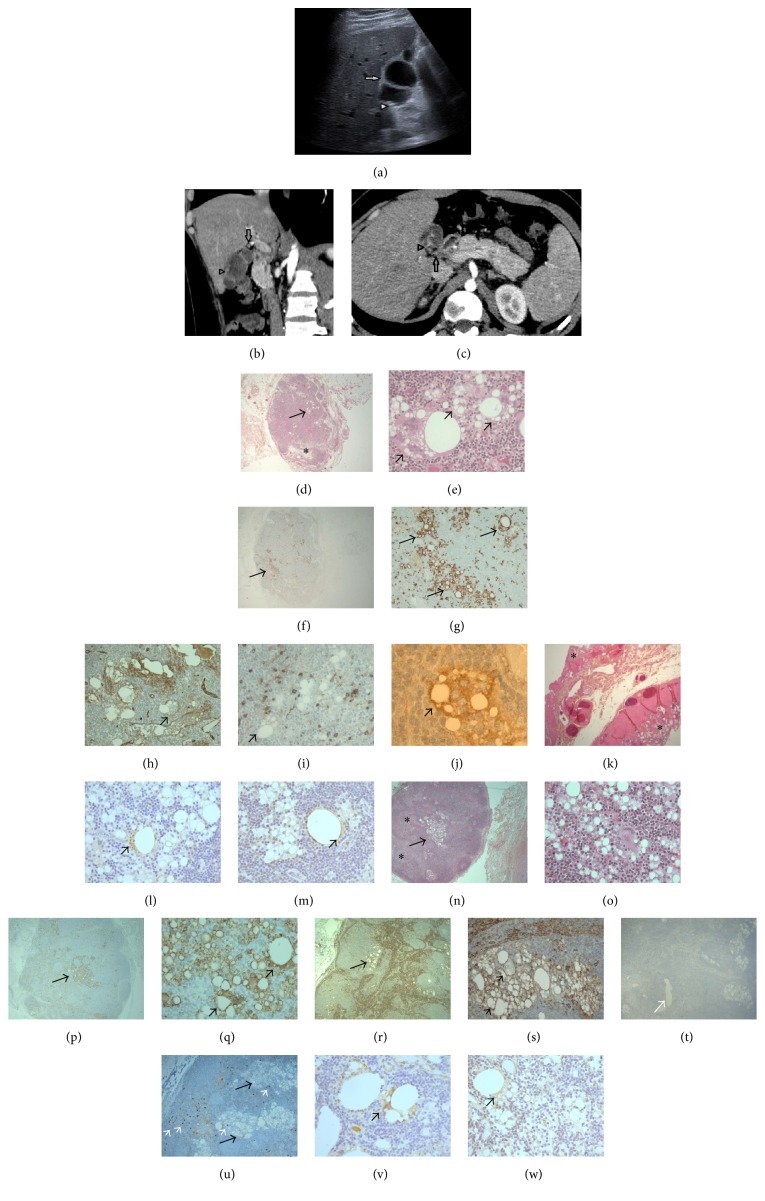
Case 1 (a–k). The ultrasound examination (a) showed irregular wall thickenings of the gallbladder wall, suggestive of septa (arrow) and lithiasis (arrowhead). On the computed tomography scan, a small lymph node was situated at the gallbladder neck (b, c: arrowhead). The gallbladder wall showed irregular thickenings suggestive of septa (arrow) (b: reconstruction image). On microscopy, the lymph node parenchyma showed oedema (d: asterisk), classical histiocytosis along with foci of lipid histiocytosis. The latter foci consisted of large multinucleated histiocytes with pink cytoplasm and one to multiple optically blank cytoplasmic vacuoles (d, e: arrows). These histiocytes expressed CD68 (f, g: arrows) and membrane CD31 (h: arrow) and did not express S100 protein (i). CD1a was expressed by scattered vacuolated cells (j: black arrow positive cells). The gallbladder septum consisted in gallbladder mucosa, submucosa, muscle layer, and adventitial tissues without fibrosis or inflammation (k: asterisks for mucosa). Adipophilin was expressed in the cytoplasm of some vacuolated cells as well as TIP47 (l and m, resp.: arrows). Case 2 (n–w). The lymph node parenchyma contained several foci of classical histiocytosis (n: asterisk for classical histiocytosis). Some histiocytes showed optically blank intracytoplasmic vacuoles (n: arrow, o). They expressed CD68 (p, q: arrows for positive cells) and membrane CD31 (r, s: arrows for positive cells). Several vacuolated histiocytes were located outside the lymph node sinuses (s). The vacuolated histiocytes did not express podoplanin (expressed in large intranodal vessel: white arrow, t). Sparse CD117-positive mastocytes were seen (u: mastocytes/white arrows, vacuolated histiocytes/black arrows). Adipophilin was expressed in some vacuolated cells while the TIP47 staining was only faintly expressed (v, w, resp.: arrows). Original magnification ×2.5 (d, f, p, t), ×5 (k, n, r, u), ×10 (g, h, i, q, s), ×20 (e, o), and ×40 (j, l, m, v, w).

One of the patients was a man (24 years old) and the other a woman (43 years old). The 1st patient presented with acute abdominal pain and elevated liver tests and lipases. The patient's BMI was 30.9. The ultrasound examination and the computed tomography scan (CT scan) (without and with iodine contrast substance injection) showed gallbladder microlithiases and irregular parietal thickenings suggestive of septa. On CT scan, the pancreas was increased in size. There were several infracentimetric mesenteric adenomegalies, a right cortical kidney lithiasis, and important gas artifacts (Figure 1). The patient's history revealed appendectomy and tonsillectomy. He did not show alcohol intake and showed smoking habits (15 cigarettes/day). The 2nd patient presented with right abdominal pain and liver tests increased in a fluctuant way. Ultrasound examination showed gallbladder lithiasis. The medical history revealed a pharyngeal papilloma, colon low-grade adenoma, bronchiolitis (smoking habits, 40 packs a year), appendectomy, depression, benfluorex-related aortic insufficiency, 2 unexplained Quincke's oedema episodes (flower shop employee), type 2 diabetes, and dyslipidemia (rosuvastatin treatment). Liver steatosis was diagnosed 2 years previously on CT scan performed without and with Omnipaque 350 injection. The patient's BMI was 35.2.

Cholecystectomy was performed for gallbladder lithiasis in both cases. The resected gallbladder measured 6 cm and contained 2 gallstones (0.4 cm) for the 1st case. For the 2nd case, the resection specimen consisted of 8 tissue fragments (2.5–4 cm) and several black gallstones (0.5 cm). Histological examination revealed, for both gallbladders, subacute and chronic lithiasic cholecystitis, with cholesterolosis in the 2nd case (Figure 1). A parietal intraluminal septum was observed in the 1st case. In both cases, a lymph node (7 and 5 cm in size, resp.) was detected at the cystic duct resection site. The lymph node parenchyma showed nodular histiocytosis. Several multinucleated giant cells containing optically blank intracytoplasmic vacuoles of varied size were also observed (Figure 1). Rare fine fibrous tractus were present as well as sparse eosinophilic polymorphonuclears and CD117 positive mast cells. Focal oedema was also observed. The histiocytic cells with vacuolated cytoplasm expressed CD68 and CD31 on immunohistochemistry. Rare vacuolated and nonvacuolated cells showed intracytoplasmic adipophilin positive granulations. TIP (tail interacting protein) 47 was also expressed in rare cells, mildly. CD1a was expressed in sparse Langerhans or related/indeterminate cells. Nuclear and cytoplasmic S100 protein was expressed in nonvacuolated histiocytic cells (possibly reactive). The vacuolated histiocytes did not express cytokeratin AE1/AE3 or D2.40 nor contain Alcian-blue-positive vacuoles. Many vacuolated cells were extravascular (CD31 and D2.40 stains). There were no S100 protein positive normal adipocytes in the lymph node parenchyma.

## 3. Discussion

Lymph node lipid histiocytosis (LH) is rarely reported nowadays. Here we report two cases in which such lesions were diagnosed at microscopic examination of gallbladder surgical resections. The histogenesis of LH is matter of debate. Lipogranulomata in human tissues such as the liver, spleen, and abdominal lymph nodes are reported to occur not only after mineral oil ingestion but also in relationship with lipemia in diabetes mellitus or associated with hepatic steatosis [4]. In the cases we present, iodinate contrast substance injection was used for the CT scans. One of the patients was diagnosed with liver steatosis on imaging procedures. Whether adipose involution/fatty replacement in the context of weight gain/obesity [5] may result in peculiar aspects of reactive histiocytosis, related or not to contrast-substance injections during imaging procedures, remains matter of debate. However, we did not observe fatty replacement of the lymph node but rather reactive lesions such as focal oedema, rare eosinophilic polymorphonuclears, and CD117-positive mast cells.

The diagnosis of LH may be difficult on HE stained tissue sections when multinucleated giant cells lack. The affected lymph nodes may be normal in size but with spongious appearance [6, 7]. However, when intraparenchymal and focal in a normal-sized lymph node, such lesions are difficult to identify on the fresh resected gallbladder specimen if not analysed on cut-section. After fixation, although these lesions are reported to be identified independently of the type of fixative solution (Stieve, Dubosq-Brasil, or formalin as in the cases we report), the diagnosis is rendered difficult by the scarcity of technical procedures for lipid detection on routinely processed tissue section [6–8]. Only frozen section examination for fat stains may identify lipids [7]. Adipophilin (perilipin-2) and TIP47 (perilipin-3) immunohistochemistries may be helpful by distinguishing cytoplasmic lipid droplets from air, as seen in the cases we report in which positive granulations were detected around cytoplasmic vacuoles [9]. However, since gallbladder neck lymph nodes are frequently not entirely analysed on microscopy and for such stains, uncertainty may persist on the true nature of the intracytoplasmic vacuoles. Of interest might be classical morphological indicators such as the presence of a fibrotic reaction and the histiocyte size, which have been proposed for the differentiation between mineral oil- and lipiodol-droplet related lesions [10]. Pneumatosis can be associated as pneumolipid histiocytosis, although probably rarely. In the cases we report, there was no biopsy, salpingo- or lymphography or surgical procedures, or radium or radiotherapy done, known to result in such lesions, however, in less than 10% of axillary or pelvic lymph nodes [6–8].

The presence of vacuolated mononuclear histiocytic cells, possibly with a signet ring morphotype as in signet ring sinus histiocytosis, may suggest a metastatic adenocarcinoma, which can be ruled out by the lack of cytokeratin expression and by the presence of Alcian blue positive mucus vacuoles. Matter of debate remains also the precise origin of the vacuolated cells despite a detailed/extensive immunohistochemical profiling. The presence of such cells both inside and outside the sinuses and the CD68+/CD31+/S100/podoplanin immunophenotype suggest rather a histiocytic than reticular or endothelial origin [11, 12]. Expression of CD1a seen in scattered vacuolated cells suggests that reactive Langerhans or indeterminate cells may also undergo cytoplasmic vacuolar change.

In conclusion, we report lipid histiocytosis in the gallbladder neck lymph nodes, incidentally diagnosed on microscopy in two obese patients having gallbladder resection for lithiasis. Etiology of such lesions is probably multifactorial, endo-, and exogeneous, prevention being possible by considering an altered vascularity (e.g., diabetes) and/or the contrast substance characteristics. The diagnosis may be difficult: LH may remain unidentified on imaging procedures while the microscopic analysis may require special stains and immunohistochemistry for ruling out an adenocarcinoma metastasis, when multinucleated giant cells are absent.
